# Comparison between Normalised and Unnormalised 454-Sequencing Libraries for Small-Scale RNA-Seq Studies

**DOI:** 10.1155/2012/281693

**Published:** 2012-01-26

**Authors:** Robert Ekblom, Jon Slate, Gavin J. Horsburgh, Tim Birkhead, Terry Burke

**Affiliations:** ^1^Department of Ecology and Genetics, Uppsala University, Norbyvägen 18 D, 75236 Uppsala, Sweden; ^2^Department of Animal and Plant Sciences, University of Sheffield, Sheffield S10 2TN, UK

## Abstract

Next-generation sequencing of transcriptomes (RNA-Seq) is being used increasingly in studies of nonmodel organisms. Here, we evaluate the effectiveness of normalising cDNA libraries prior to sequencing in a small-scale study of the zebra finch. We find that assemblies produced from normalised libraries had a larger number of contigs but used fewer reads compared to unnormalised libraries. Considerably more genes were also detected using the contigs produced from normalised cDNA, and microsatellite discovery was up to 73% more efficient in these. There was a positive correlation between the detected expression level of genes in normalised and unnormalised cDNA, and there was no difference in the number of genes identified as being differentially expressed between blood and spleen for the normalised and unnormalised libraries. We conclude that normalised cDNA libraries are preferable for many applications of RNA-Seq and that these can also be used in quantitative gene expression studies.

## 1. Introduction

Next-generation sequencing (NGS) has revolutionised biological research and opened up the field of genomics for small-scale projects in nonmodel organisms [1–3]. A practical approach for studies of species with no prior genomics information available, and where budgets are limited, is to sequence only the expressed parts of the genomes (transcriptomes). This method, known as RNA-Seq [4], has the advantage that sequence characterisation is focused on functionally important regions of the genomes. An additional benefit is that information is obtained not only about gene sequence variation but also regarding gene expression levels [5]. While most publications on transcriptome characterisation in nonmodel organisms remain rather descriptive, question-oriented papers are also emerging in a number of fields, including speciation [6], conservation [7], and local adaptation [8]. The Roche 454 sequencing technology is probably still the most widely used NGS method for de novo characterisation of transcriptomes of nonmodel organisms, but other methods such as Illumina/Solexa and ABI SOLiD are becoming increasingly popular [9, 10].

Data from small-scale RNA-Seq studies are routinely used for a number of different purposes such as gene finding, marker identification, and expression studies [1]. Data from related species, genomic reference species [11], are often utilised to annotate whole-transcriptome sequence datasets or to identify specific genes of interest. Molecular markers such as microsatellites, indels, and SNPs can be efficiently mined from NGS transcriptome datasets, as reported in a number of recent publications (e.g., [12–15]). RNA-Seq studies can also address questions about differential expression between for example different tissues, life stages, individuals or populations, differences that may be important for understanding gene function, development, phenotypic plasticity, local adaptation and speciation [6, 16, 17]. Even small amounts of sequencing, using only a fraction of a sequencing run, may enable these aims to be achieved efficiently in nonmodel organisms. But it is important to plan the sequencing effort carefully in advance to invest optimally in a methodology that will enable the posed questions to be answered. 

Several methods exist for improving transcriptome data in order to get a more even coverage of genes and to avoid spending a large part of the sequencing effort on a few very highly transcribed genes. The most common of these is the duplex-specific nuclease (DSN) normalization procedure [18], but other cDNA normalisation methods are also available [19]. In general, cDNA normalisation is applied in studies in which the main aim is to characterise as many genes as possible or when using the sequence data to identify molecular markers. Unnormalised libraries are instead mainly used to characterise gene expression levels or investigate differential expression between samples. The normalisation of cDNA libraries for NGS has previously been evaluated in the lake sturgeon (*Acipenser fulvescens*). Here, Hale and coworkers [20] demonstrated, using rarefaction analysis, that normalisation has only a rather limited influence on improving gene discovery, provided that a large enough number of sequence reads are available. Gene discovery efficiency was also compared between normalised and unnormalised 454-sequenced cDNA libraries of bitter melon (*Momordica charantia*) seeds [21]. Here, it was found that normalised libraries produced more and longer contigs compared to unnormalised libraries and that, in contrast to the results of Hale et al., normalisation considerably enhanced the rate of gene discovery. These results may however be biased since the number of sequencing reads produced from the unnormalised library was almost twice as large as the number of reads from the normalised library. An increase in gene discovery in normalised compared to unnormalised libraries has also been reported from a study of milkweed bug (*Oncopeltus fasciatus*) embryos [22]. An alternative to cDNA normalisation is to specifically remove only rRNA [1], which may represent a large fraction of the RNA in a sample. Another way to increase the sequencing efficiency and quality of NGS cDNA libraries may be to remove specifically the poly(A) tails prior to sequencing, using restriction enzymes [23].

The aim of this study was to compare the efficiency of sequencing transcriptomes, using the 454-technology, from normalised and unnormalised cDNA libraries in a small-scale RNA-Seq study of two zebra finch (*Taeniopygia guttata*) tissues (blood and spleen). We consider several downstream applications such as gene discovery, differential expression studies and microsatellite marker identification. In order to conduct an unbiased comparison between normalised and unnormalised libraries we compare sequence datasets that have identical numbers of reads and read-length distributions. This is accomplished by drawing subsamples of reads from the full-read datasets and conducting de novo assemblies and downstream analyses on these subsamples separately.

## 2. Methods

### 2.1. Library Preparation and Sequencing

Blood and spleen tissue samples were obtained from one adult zebra finch male from the University of Sheffield captive colony [24] and immediately stored in RNAlater (Ambion Inc.). RNA was extracted from the samples using the RNeasy kit (QIAGEN), and cDNA was synthesised using the MINT kit (Evrogen). cDNA was purified through QIAquick PCR Purification columns (QIAGEN) and diluted to a concentration of ~100 ng/*μ*L (measured on a Nanodrop, Thermo Scientific). Half of the cDNA from both blood and spleen was normalised using the Trimmer kit (Evrogen, [18]). Briefly, cDNA was mixed with hybridisation buffer and heated to 98°C for 2 minutes and then to 68°C for 5 hours. DSN (Duplex Specific Nuclease) master buffer and dilutions of the DSN enzyme were added and incubated at 68°C for 25 minutes. A control containing no DNS enzyme was also included. Reactions were stopped by adding DSN stop solution, and samples were kept on ice. The primary amplification of the cDNA was performed according to the manufacturer's protocol, and aliquots were taken at two-cycle intervals. Each of the aliquots was run on a 1% agarose gel to determine the respective cycle numbers during which the PCR was still in its exponential phase; 22 cycles were chosen for blood and nine cycles for spleen.

**Figure 1:**
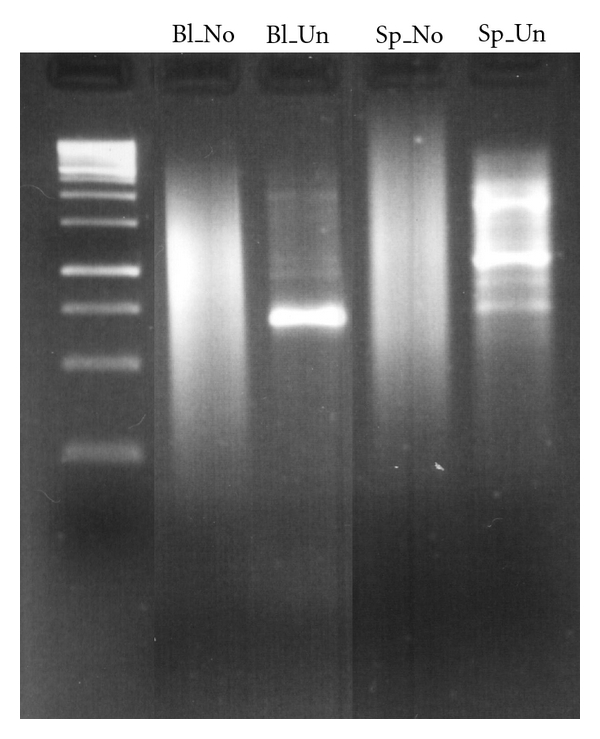
Electrophoresis (in 1% agarose) of the sequenced cDNA libraries. Bl: blood, Sp: spleen, Un: unnormalised, and No: normalised. The ladder in the leftmost lane has the following band sizes (in bp from below): 250, 500, 750, 1000 (bright), 1500, 2000, 2500, 3000 (bright), and 3500–10000 (smear).

Samples obtained using these optimal numbers of cycles were again run on a 1% gel to verify the effect of normalisation (Figure 1) and to determine the best DSN treatment concentration. The samples with the least DSN were selected and subjected to a secondary amplification. The resulting cDNA libraries were sent for 454 sequencing (Roche, FLX) at the Centre for Genomic Research, University of Liverpool (http://www.liv.ac.uk/cgr/index.html). Each sample was sequenced on 1/8th of a 454-sequencing plate. The raw read data from the sequencing were deposited in the NCBI sequence read archive under project accession number SRP003283.1.

### 2.2. Sequence Analysis and Assembly

Raw 454-sequence reads were trimmed of low-quality sequence, adaptor and primer sequence and poly(A) tails using SeqMan NGen version 2.0 (DNASTAR, Inc.). In order to compare the efficiency of reads from different libraries (normalised and unnormalised), we randomly subsampled (without replacement) ten sets of sequences from each library with identical numbers of reads (29500 for blood and 45800 for spleen) and distributions of read lengths (from 30 to 499 base pairs), for both the normalised and the unnormalised libraries. The read-length distribution used was the same as that in the full sequence data. This jackknifing procedure was performed to create comparable estimates of the mean of each relevant metric in the downstream analyses. Each of the 40 subsampled sequence sets was then independently de novo assembled using the default settings in Newbler (Roche, gsAssembler version 2.0).

### 2.3. Gene Discovery

All contigs produced by the 40 de novo assemblies (ten subsampled sequence data sets from each of normalised blood, unnormalised blood, normalised spleen, and unnormalised spleen libraries) were blasted (BLASTN version 2.2.17) [25] against the chicken gene predictions (WASHUC 2.57) downloaded from the Ensembl [26] FTP site. Only the best hit per contig and only hits with an *e*-value less than 10^−5^ were kept. Numbers of overlapping genes between libraries were calculated using the LIMMA library implemented in R [27, 28] and visualised in a Venn diagram.

### 2.4. Expression Analysis

The number of reads per gene was calculated by summing the number of reads for each of the contigs that had a best blast match for the gene in question (often more than one contig matched the same gene, presumably due to incomplete coverage of genes and possibly also representing different alleles or isoforms). Differential expression between blood and spleen was assessed using the TMM normalisation procedure included in the edgeR, Bioconductor package [29, 30]. Genes were defined as being differentially expressed in the two tissues if they had a probability of less than 0.05 after adjusting for multiple testing using the Benjamini and Hochberg [31] method for controlling the false discovery rate (FDR).

### 2.5. Microsatellite Discovery

The contigs produced from all subsampled sequence sets were independently searched for microsatellite repeats using the software MsatCommander [32]. A microsatellite was called if the contig contained a motif that was repeated for at least six units for dinucleotides and at least four units for tri-, tetra-, penta-, and hexanucleotides. When comparing the microsatellite discovery efficiency between normalised and unnormalised libraries, the numbers of all types of repeats (di-, tri-, tetra-, penta-, and hexanucleotides) were summed for each subsampled sequence set separately.

### 2.6. Statistical Analyses

All statistical analyses and handling of large output data files were conducted in R version 2.11.1 [28]. All reported significance tests are two-tailed.

## 3. Results and Discussion

### 3.1. Sequencing

In total, 478,888 raw reads were produced by 454 sequencing (Table 1). For normalised and unnormalised libraries from blood we obtained in total 73,602 and 119,770 reads, respectively, while 117,710 and 167,806 reads were produced from the normalised and unnormalised spleen libraries. After quality trimming and removal of primer, adaptor, and poly(A) sequences, 393,722 sequence reads remained (Table 1). From the normalised blood library there were 59,616 trimmed reads with a mean read length of 304 base pairs, and from unnormalised blood there were 100,516 reads with a mean length of 352 base pairs. There were 86,323 reads (mean length = 281 base pairs) available for the normalised spleen library and 147,267 reads (mean length = 301 base pairs) for unnormalised spleen. Our unnormalised libraries thus produced more sequence data than normalised libraries. The unnormalised data were also of higher quality since a smaller proportion of the reads was removed during trimming and the resulting trimmed reads were longer (Table 1). These differences could simply be due to random events in the sequence reactions and plate partitions used, but it is seems more likely that they represent some intrinsic properties of the library treatments since similar metrics have also been reported from other studies [20–22].

### 3.2. De Novo Assembly

In order to make direct and unbiased comparisons between normalised and unnormalised cDNA libraries, we estimated the mean of each relevant metric from ten subsamples of sequences (with identical numbers of reads and read length distributions) from each library. These were assembled separately, and the contigs from these assemblies were used in downstream analyses. De novo assemblies of reads produced from normalised libraries had a larger number of contigs but used fewer of the reads compared to assemblies from unnormalised libraries (Table 2). Contigs from unnormalised libraries were also shorter than normalised library contigs (Table 2), with both smaller mean (first subsample of blood:  *t* = 6.8, *df* = 1473, *P* < 0.0001; first subsample of spleen: *t* = 9.1, *df* = 2713, *P* < 0.0001) and maximum contig lengths. We also found strikingly similar results using our full dataset, even though this included a much higher number of reads (and with higher quality, see the previous paragraph) for unnormalised than normalised libraries (Table 1).

**Figure 2:**
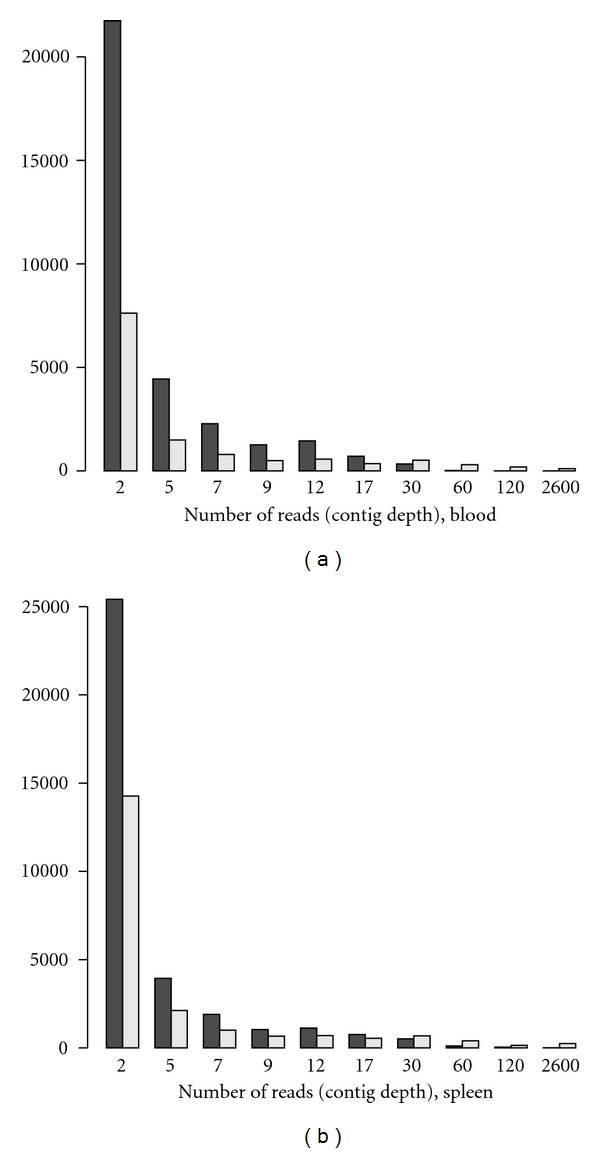
The distributions of the depths of contigs (*y*-axis: number of reads) for (a) blood and (b) spleen. Black bars represent number of contigs from normalised libraries, and grey bars represent number of contigs from unnormalised libraries. The mean position of the bins is given on the *x*-axis.

The maximum number of reads per contig (contig depth) was lower for normalised libraries than for unnormalised (Table 2) and so was the mean contig depth (first subsample of blood: *t* = 3.8, *df* = 1241, *P* < 0.001; first subsample of spleen: *t* = 5.1, *df* = 2140, *P* < 0.0001). A large majority of contigs from normalised libraries had a contig depth of only a few reads. At the other extreme several contigs from each subsampled unnormalised library had more than a thousand reads (Figure 2). With all reads included in the assemblies the mean number of reads per contig was 9.3 for normalised libraries and 31.0 for unnormalised. Similar observations to these were made when assemblies from normalised and unnormalised cDNA were compared in milkweed bug [22].

### 3.3. Gene Discovery

**Figure 3:**
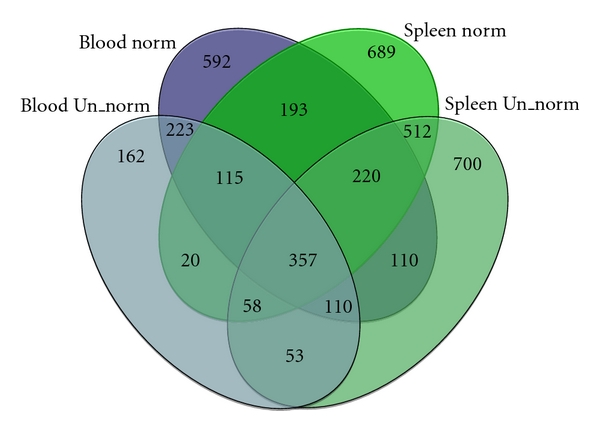
Venn diagram showing the number of genes identified in the different sequenced libraries. Blue areas represent sequences from blood and green sequences from spleen. Dark colours represent sequences from normalised libraries, and light colours represent sequences from unnormalised libraries. Numbers in overlapping areas represent genes identified in more than one library.

We annotated the contigs for all subsampled sequence sets separately by comparing them to annotated transcripts in the chicken genome. Considerably more genes (transcripts) were detected in the contigs produced from normalised cDNA libraries compared to unnormalised library contigs (Table 2). This was true both for sequences from blood and spleen (Figure 3). However, the mean number of reads for each gene was larger for the unnormalised libraries (Mann-Whitney test; blood: *U* = 1.9 × 10^6^, *df* = 4113, *P* < 0.0001; spleen: *U* = 2.8 × 10^6^, *df* = 4113, *P* < 0.0001, Table 2). The highest expression in the normalised blood library was for the *ATRX* gene, with up to 85 reads per subsampled sequence library. In contrast, two genes (*Haemoglobin alpha-A* and *-B*) were represented by over a thousand sequence reads in the unnormalised blood library. By far the most highly expressed gene in the unnormalised spleen library was a mitochondrial rRNA gene, with over five thousand reads present. In the normalised spleen library the gene with the highest expression (*Reticulocalbin-2*) had only a maximum of 90 reads present. These results are in contrast to one previous study, in which normalisation of the cDNA library did not enhance gene finding in a 454-sequenced transcriptome dataset of the milkweed bug [22]. Similarly, gene finding efficiency was only marginally improved by normalisation of the libraries in the sturgeon [20]. Differences among the results in these studies may be due to differences in the degree of sequencing effort. As the total number of sequence reads increases, the difference in gene-finding efficiency between normalised and unnormalised libraries should become smaller.

### 3.4. Expression Analysis

**Figure 4:**
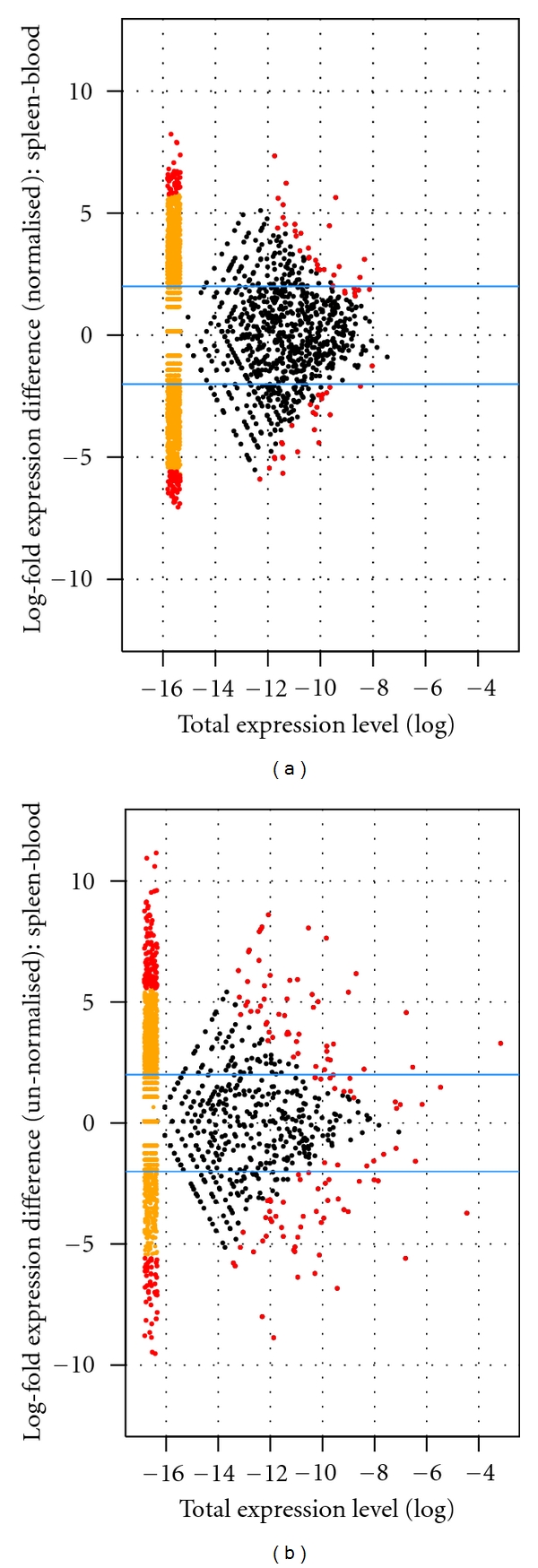
Differential expression of genes between spleen and blood, data from (a) normalised cDNA libraries and (b) unnormalised libraries. Positive log-fold differences indicate higher expression in spleen than in blood. The blue horizontal lines represent fourfold differences in expression between tissues. Genes expressed in only one tissue are plotted in yellow smears to the left of the graphs. Red points represent genes showing significant expression divergence between tissues (*P* < 0.05, after applying an FDR multiple test correction).

Most RNA-Seq studies of gene expression levels have used unnormalised cDNA libraries in order to introduce as little bias as possible in the relative abundance of sequence reads from different genes. However, given that there is enough variation in cDNA levels remaining after normalisation and that the relative normalised read abundance is positively correlated with the original expression level, such studies can also be conducted on normalised libraries [33, 34]. We found a highly significant positive correlation between the detected expression level (log number of reads) of genes between normalised and unnormalised libraries (blood: *r* = 0.36, *df* = 803, *P* < 0.0001; spleen: *r* = 0.39, *df* = 1145, *P* < 0.0001). In a previous study Kristiansson and coworkers [17] similarly found a strong correlation between expression level (in this case measured by a microarray approach) and number of 454 reads from a normalised cDNA library. In our data there was also a weak correlation between the detected expression levels of genes between the two different tissues sampled (unnormalised library: *r*_*S*_ = 0.13, *df* = 4112, *P* < 0.0001; normalised library: *r*_*S*_ = 0.07, *df* = 4112, *P* < 0.0001). Means of 311 and 328 genes (7.6 and 8.0 per cent of all genes) were differentially expressed between blood and spleen for normalised and unnormalised libraries, respectively (Figure 4). Nonetheless, as expected, genes from the unnormalised libraries had a larger maximum log-fold difference in expression compared to genes in the normalised library (Figure 4).

### 3.5. Microsatellite Discovery

NGS transcriptome data has been extensively utilised for identifying microsatellites, also known as SSRs (simple sequence repeats), in nonmodel organisms (e.g., [12, 35, 36]). Repeat motifs are usually present in the untranslated regions of the transcripts [37]. Normalised cDNA libraries seem to be very effective for this application, providing a broad representation of the transcriptome. But a formal comparison between the efficiency of normalised and unnormalised libraries for identifying microsatellites has, to the best of our knowledge, not previously been performed. Using our whole dataset (including contig sequences produced using all sequence reads from both tissues), in total 502 microsatellite repeats (dinucleotides to hexanucleotides) were found (318 from normalised and 184 from unnormalised libraries) with the software MsatCommander [32]. When comparing the ten subsampled datasets from each library, we found, on average, 66% more microsatellites in normalised blood libraries and 73% more in normalised spleen libraries than in the unnormalised libraries (Table 3). Since our sequence data were obtained from one single individual and the coverage of contigs was rather low, we did not address the level of variation in the identified markers. However, in other cases where microsatellites have been identified using this approach, around 50% of the markers have been found to be amplifiable and polymorphic in validation studies [13, 38], suggesting that this method is very efficient for finding useful molecular markers. Another advantage of this method of identifying molecular markers is that variation can often be associated with annotated genes, facilitating interpretation of outlier loci and candidate genes for adaptation [1].

## 4. Conclusions

In contrast to some previous studies we find a much higher efficiency of gene discovery when using normalised cDNA libraries compared to unnormalised libraries in RNA-Seq studies. The normalised libraries were also more efficient for finding microsatellite markers. We also demonstrate that normalised cDNA can be used in characterising expression variation due to a correlation between the relative number of reads per gene in the contigs from normalised and unnormalised libraries. Some of these results are different from those of other studies, concluding that gene-finding efficiency is only marginally improved by normalisation [20, 22]. It is hard to speculate about the exact causes of these discrepancies but differences in technologies used and sequencing efforts are both likely to affect the outcome of this kind of comparison. The results from our study are mainly applicable to small-scale investigations of nonmodel organisms. However, as sequencing technologies continue to improve and the cost of sequencing drops further, even small-scale studies may be able to produce high enough coverage of transcriptomes to make the normalisation procedure superfluous.

## Figures and Tables

**Table 1:** Summary of output from 454 sequencing (full dataset) and statistics from the full data de novo assemblies.

	Normalised	Unnormalised
Number of raw reads	191312	287576
Number of reads after trimming	145939	247783
Percent of reads retained	76.3	86.2
Mean read length after trimming	290	322
Number of contigs in assembly	5563	2703
Mean contig length (bp)	660	865
Maximum contig length (bp)	1412	4382

**Table 2:** Summary of statistics (means with standard errors estimated through jackknifing) of contigs produced by de novo assembly of 454-sequencing reads from normalised and unnormalised transcriptome libraries.

	Blood		Spleen	
	Normalised	Unnormalised	Normalised	Unnormalised
Number of sampled reads	29500 (0)	29500 (0)	45800 (0)	45800 (0)
Number of assembled reads	13998 (89.0)	20703 (25.9)	15980 (29.6)	22487 (23.3)
Number of contigs in assembly	3221 (6.1)	1240 (7.4)	3487 (12.8)	2078 (10.6)
Mean contig length	413.7 (0.8)	501.6 (1.8)	385.7 (0.6)	455.2 (1.2)
Maximum contig length	2014 (105)	4777 (240)	1428 (15)	3117 (54)
Mean number of reads per contig	4.7 (0)	13.4 (0.2)	4.7 (0.05)	14.2 (0.3)
Maximum number of reads per contig	48 (1.1)	1944 (108.2)	230 (3.8)	1944 (165.8)
Number of transcripts detected	1184 (5.3)	586 (14.7)	1238 (5.9)	924 (6.0)
Mean number of reads per transcript	7.1 (0.04)	23.9 (0.6)	6.6 (0.03)	22.0 (0.5)

**Table 3:** Number of microsatellite repeats found in contigs produced from ten subsampled datasets of normalised and unnormalised zebra finch transcriptome libraries (means with standard errors estimated through jackknifing).

Repeat type	Blood		Spleen	
	Normalised	Unnormalised	Normalised	Unnormalised
Dinucleotide	17.8 (0.9)	7.8 (0.9)	30.4 (1.3)	10.8 (0.5)
Trinucleotide	60.1 (1.6)	39.1 (1.4)	72.6 (1.5)	52.5 (1.6)
Tetranucleotide	6.5 (0.3)	3.7 (0.3)	12.5 (0.7)	5.5 (0.5)
Pentanucleotide	0.1 (0.1)	0.2 (0.1)	3.5 (0.5)	0.2 (0.1)
Hexanucleotide	0 (0)	0.1 (0.1)	1.0 (0.1)	0.2 (0.1)

Total	84.5 (1.2)	50.9 (2.0)	120.0 (2.6)	69.2 (1.5)
